# AWARE-Net: A Lightweight Joint Optimization Framework for Robust Sensor-Based Human Activity Recognition

**DOI:** 10.3390/s26144566

**Published:** 2026-07-18

**Authors:** Pei He, Yuyan Wang, Pengxin Ren, Xiaodong Wang, Lishuai Xie, Yangming Guo

**Affiliations:** 1 School of Computer Science, Northwestern Polytechnical University, Xi’an 710072, China; hepei002@mail.nwpu.edu.cn (P.H.);; 2 College of Computer and Information Science, Guangdong University of Technology, Guangzhou 510006, China; 3 Ningbo Institute of Northwestern Polytechnical University, Ningbo 315103, China; 4 Jiangsu Key Laboratory of Advanced Structural Materials and Application Technology, Nanjing Institute of Technology, Nanjing 211167, China

**Keywords:** sensor-based human activity recognition (SHAR), lightweight deep learning, class-balanced loss, contrastive learning, total variation regularization

## Abstract

Sensor-based human activity recognition (SHAR) serves as a core research direction in pervasive computing, mobile health, and related fields. Although existing deep learning methods have achieved promising progress in SHAR tasks, most optimize from a single dimension only. They struggle to simultaneously balance recognition accuracy, noise robustness, adaptation to class imbalance, and lightweight deployment requirements, leading to performance bottlenecks in real-world scenarios. To address these challenges, this paper proposes a lightweight joint optimization framework named AWARE-Net. Leveraging the lightweight TS-ResNet as a backbone encoder, the framework integrates spatiotemporal dynamic convolution feature encoding with a global loss function that fuses class-balanced loss, contrastive learning auxiliary loss, and temporal smooth regularization to achieve multi-objective joint optimization. Extensive experiments on three widely used SHAR benchmark datasets, namely OPPORTUNITY, PAMAP2, and USC-HAD, demonstrate that the proposed AWARE-Net achieves competitive performance compared with representative state-of-the-art HAR methods.

## 1. Introduction

Sensor-based human activity recognition (SHAR) is a fundamental sensing technique for pervasive computing, mobile health, smart homes, and intelligent security [1,2]. Using inertial sensors embedded in wearable devices, SHAR aims to model human motion time-series signals and automatically infer daily or task-related activity patterns. Its core goal is not merely to improve the performance of a single feature extraction or classification module but to achieve stable recognition, accurate prediction, and efficient deployment at the overall system level.

However, SHAR remains challenging due to the coupled effects of noisy sensor signals, imbalanced activity distributions, and model complexity. Wearable sensor signals are often affected by sensor-placement shifts, individual motion differences, motion artifacts, and environmental interference, which may weaken discriminative temporal patterns. Meanwhile, minority or transitional activities are more likely to be misclassified under noisy sensing conditions. Although increasing model capacity may improve representation ability, it can also introduce additional computational cost. Therefore, the key problem is to learn robust and discriminative activity representations from noisy and imbalanced sensor sequences while maintaining a compact and efficient model structure [3].

Existing SHAR studies have mainly advanced along two directions: architecture design and training optimization. At the model architecture level, based on Convolutional Neural Networks (CNNs) [4,5] and Long Short-Term Memory (LSTM) [6,7], researchers have introduced modules such as self-attention mechanisms [8,9], dual-path attention fusion, and dynamic convolution, which significantly improve a model’s feature representation capability for complex time-series signals. On the other hand, to adapt to the limited computing power and storage resources of wearable devices, lightweight designs such as depthwise separable convolution [10,11], adaptive channel pruning, and multi-resolution residual attention have been widely adopted, which minimize the reduction in recognition performance while compressing model parameters and computational costs. At the training optimization strategy level, existing studies have proposed targeted solutions for noise robustness and class imbalance. For sensor noise interference, contrastive learning [12,13], adaptive temporal weighting, and other methods are used to enhance the invariance of features to low-level disturbances and improve the cross-scenario generalization ability of the model. For long-tailed data distributions, strategies including SMOTE oversampling [14], class-balanced loss [15], and cost-sensitive ensemble learning are adopted to alleviate the model’s training bias toward majority classes and improve the recognition performance of minority activities [16]. However, most existing methods address these issues from a single perspective. Architecture-oriented methods mainly enhance feature extraction, while optimization-oriented methods usually target one specific problem, such as noise robustness or class imbalance. In SHAR, these factors are coupled: Noisy signals may further obscure minority-class patterns, and improving representation abilities with a larger model may increase computational cost. Therefore, a more unified framework is needed to jointly consider robust representation learning, class-imbalance-aware optimization, and model compactness.

Motivated by the above observations, this paper proposes AWARE-Net, a lightweight reliability-aware framework for SHAR under noisy and class-imbalanced conditions. AWARE-Net adopts a compact TS-ResNet-based spatiotemporal encoder to capture dynamic sensor patterns with low computational complexity. In addition, a joint optimization objective is designed by integrating class-balanced supervised classification, SimCLR-based contrastive representation learning, and total variation (TV) regularization. The class-balanced term reduces the bias toward majority activities, the contrastive term enhances feature robustness under disturbed sensor signals, and the TV regularization term improves temporal consistency within each sliding window. Through this design, AWARE-Net aims to achieve a better balance among recognition accuracy, robustness, class-imbalance handling, and model compactness.

Experiments are conducted on three public SHAR benchmark datasets to evaluate the proposed method. The results show that AWARE-Net achieves competitive or superior performance compared with representative HAR methods across multiple evaluation metrics. Meanwhile, with only 1.18 M parameters and 0.01 GFLOPs, AWARE-Net maintains a small model size and low computational complexity, indicating its potential suitability for resource-constrained applications. The contributions of this work can be summarized as follows:

- We propose AWARE-Net, a lightweight reliability-aware SHAR framework that combines a compact spatiotemporal feature encoder with joint optimization to address noisy sensing, class imbalance, and model compactness in a unified manner.
- We design a unified training objective that integrates class-balanced supervised classification, SimCLR-based contrastive representation learning, and TV regularization. This objective reduces majority-class bias, improves feature robustness, and enhances temporal consistency within sliding windows.
- We conduct extensive experiments on three public SHAR benchmark datasets. The results show that AWARE-Net achieves competitive or superior recognition performance with a small model size and low computational complexity, and ablation studies validate the contribution of each component.

The remainder of this paper is organized as follows. Section 2 reviews related work on spatiotemporal feature modeling and lightweight design in SHAR. Section 3 presents the architecture of AWARE-Net and the details of its optimization strategy. Section 4 introduces the experimental settings and provides a comprehensive analysis of the results. Section 5 analyzes the computational complexity of the model and its potential for edge deployment. Section 6 concludes this paper and discusses future research directions.

## 2. Related Works

This section analyzes the core technical challenges of wearable sensor-based human activity recognition in real-world deployment scenarios. It reviews representative approaches from four key perspectives, including spatiotemporal feature modeling, lightweight design, robustness enhancement, and imbalanced data handling, and it examines their inherent limitations to clarify the positioning of this study.

### 2.1. Dynamic Spatiotemporal Feature Modeling and Lightweight Deployment Challenges in SHAR

SHAR relies on inertial time-series signals collected by wearable sensors to model human activities. In practical scenarios, these signals are often affected by variations in sensor placement, individual motion patterns, and environmental electromagnetic interference. Static networks with fixed parameters are therefore insufficient to adaptively capture dynamic spatiotemporal patterns of activities, and they tend to extract ineffective features under strong noise conditions. Meanwhile, SHAR is typically deployed on resource-constrained platforms such as wearable devices and edge terminals, where existing high-performance models still suffer from large parameter sizes and high computational costs.

Extensive efforts have been devoted to temporal modeling and lightweight design. For temporal feature extraction, recurrent networks such as LSTM [6] serve as classical baselines for SHAR. Recent works, such as MFCCT [5], further improve temporal modeling by combining spectral–temporal fusion with DeepConvLSTM [17] to robustly capture complex sequence patterns. Zeng et al. [4] improved one-dimensional CNNs by optimizing weight sharing, which significantly enhanced local feature extraction efficiency and parallel training capabilities. The residual structure in ResNet [18] mitigates the degradation problem in deep networks and becomes a mainstream backbone for SHAR feature extraction. In terms of lightweight design, MobileNetV2 [19] achieves substantial reductions in parameters and computation via depthwise separable convolutions. Further studies explore the trade-off between accuracy and efficiency through dynamic convolution, structural optimization, and adaptive channel pruning. Representative methods include TS-DyConv [20] and channel selection CNNs [21]. For instance, RepMobile [10] utilizes structural reparameterization for faster inference, while the innovative dual-decoupling CNN [22] enhances memory efficiency via layer-wise temporal–spatial attention. Recently, Petti et al. investigated robust human activity prediction through multi-view sensor data integration, addressing noise robustness and class imbalance on wearable HAR datasets such as PAMAP2 [23].

Despite these advances, existing methods still exhibit limited feature robustness under strong noise and face persistent challenges in model compression, as maintaining high discriminative power under extremely low computational budgets remains difficult.

### 2.2. Multi-Objective Optimization Strategies for Noise Robustness and Class Imbalance

In real-world SHAR scenarios, strong noise and long-tailed class distributions often coexist. Existing studies address these challenges from two main directions. For robustness enhancement, methods such as adaptive temporal attention mechanisms [24] assign differentiated weights across temporal dimensions, enabling models to focus on informative signal segments and suppress noise interference. Contrastive learning is also introduced into SHAR to improve cross-scenario generalization; for example, Li et al. [25] employ contrastive loss to enforce invariant semantic representations, and the CLEAR framework [13] utilizes supervised contrastive learning to refine feature discriminability under multimodal noise. Guo and Nakayama [26] proposed a multi-task supervised contrastive learning framework and jointly optimized activity classification and contrastive objectives to learn user-invariant and activity-discriminative representations for cross-user generalization.

To address class imbalance, prior work considers both data-level and algorithm-level strategies. At the data level, techniques such as resampling and cost-sensitive learning [14] have been employed to alleviate training bias caused by imbalanced activity distributions in wearable HAR. At the algorithm level, Buda et al. [27] systematically demonstrate the negative impact of long-tailed distributions on model training, while methods such as cost-sensitive ensemble frameworks [28] and class-balanced loss [15] improve minority class recognition through adaptive reweighting. Singh et al. [29] further provide an evaluative analysis of deep learning approaches under class imbalance, highlighting the role of model explainability in understanding prediction bias. More recently, Ameen and Aminifar [30] integrated redundancy-aware CNN pooling mechanisms with class-weighted loss to simultaneously enhance noise robustness and mitigate class imbalance in mobile sensor-based HAR.

Although these approaches enhance feature representation through attention mechanisms and mitigate training bias via optimization strategies, they are often developed from isolated perspectives. In practical SHAR scenarios, complex temporal dependencies and class imbalance coexist. It is therefore necessary to explore more coordinated modeling and optimization strategies within a unified framework to improve the overall robustness and generalization ability of models for complex sensor-based activity recognition tasks.

## 3. Methodology

In this section, we propose a lightweight joint optimization framework termed AWARE-Net, together with a series of systematic optimization strategies. We first elaborate on the formal definition and core challenges of the SHAR task and clarify the design motivation of our proposed method by dissecting the inherent limitations of existing approaches when dealing with complex SHAR scenarios. Subsequently, we provide a detailed elaboration on the overall architecture, core encoding modules, and multi-objective joint optimization strategy of the proposed AWARE-Net framework.

### 3.1. Problem Formulation and Design Motivation

#### 3.1.1. Task Formal Definition

Sensor-based human activity recognition aims to learn a mapping function from multi-channel inertial sensor time-series signals to predefined human activity categories. Specifically, we represent the original sensor dataset as a training sample set $D={\left\{\left(x_{i},y_{i}\right)\right\}}_{i=1}^{N}$, where *N* is the total number of samples, $x_{i}\in R^{T\times C}$ is the input sensor time series, *T* is the length of the time window after sliding window segmentation, *C* is the number of sensor channels (e.g., accelerometer, gyroscope, magnetometer), $y_{i}\in \left\{1,2,\ldots ,C_{cls}\right\}$ is the ground-truth activity label, and $C_{cls}$ is the total number of activity categories.

The core objective of the SHAR task is to learn an end-to-end classifier $f:R^{T\times C}\to \left\{1,2,\ldots ,C_{cls}\right\}$, which can accurately map unlabeled sensor time series to the corresponding activity labels. The standard workflow of the SHAR task is defined as follows:

(1) $$\begin{array}{c} x^{\prime }=SEGMENTATION\left(x\right)\\ h_{i}=REPRESENTATION\left(x_{i}^{\prime }\right)\\ L=OPTIMIZATION(h_{i},y_{i})\\ y_{i}=PREDICTION\left(h_{i}\right) \end{array}$$

where SEGMENTATION denotes the sliding window’s preprocessing on raw sensor data, REPRESENTATION is the feature encoding module of the model, OPTIMIZATION is the loss function for model training, and PREDICTION is the final classification inference process.

#### 3.1.2. Design Motivation

In real-world scenarios, SHAR, when using wearable sensors, suffers from several critical issues, including complex noise in sensor signals, intra-class data imbalance, and the difficulty of balancing model accuracy and lightweight deployment. Most existing methods adopt static spatiotemporal feature extraction with fixed parameters, which is difficult to adapt to spatiotemporal dynamic variations and noise disturbances of human activities, and tends to extract invalid features under high noise. They suffer from obvious prediction bias on long-tailed data, and their complex network structures cannot meet the lightweight requirements of mobile terminals. To address the above challenges, this paper proposes a lightweight joint optimization framework named AWARE-Net. It leverages dynamic spatiotemporal convolution to suppress noise and capture critical activity features. By integrating class-balanced supervised classification, SimCLR contrastive learning, and total variation smooth regularization into a unified optimization paradigm, the framework enhances robustness against strong noise and data imbalance, as well as the stability of continuous activity prediction.

### 3.2. Overall Architecture of AWARE-Net

**Figure 1:**
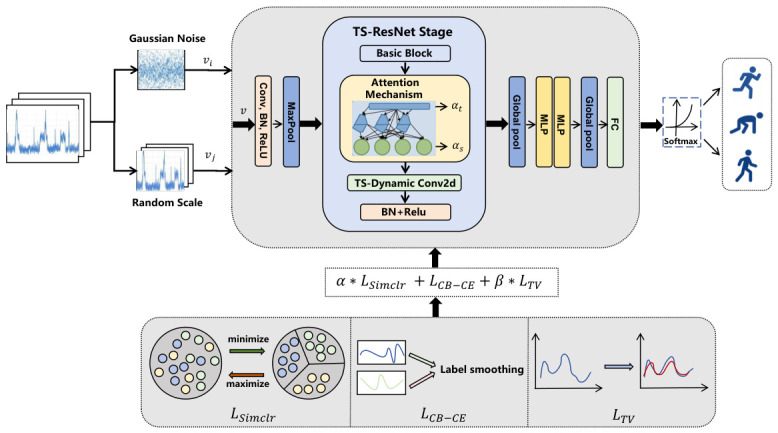
Overall architecture of the proposed AWARE-Net framework.

The overall architecture of the proposed AWARE-Net is illustrated in Figure 1, which mainly consists of a data augmentation module, a spatiotemporal dynamic residual feature encoder, and a multi-objective joint optimization loss function. Specifically, the raw sensor time series are segmented into fixed-length sequences via a sliding window. The data augmentation module independently applies two distinct random perturbations to the same input sequence, generating two augmented views with consistent semantics. These two views are concatenated along the batch dimension and then fed into the feature encoder. The input goes through an initial convolutional layer, a batch normalization layer, a ReLU activation function, and a max-pooling layer to accomplish preliminary feature extraction and dimensionality reduction. Subsequently, the features are fed into the TS-ResNet residual blocks for deep feature encoding, where dynamic weighted aggregation of multi-scale kernels is realized. The encoded deep features are passed to a global average pooling layer, followed by a two-layer MLP projection head and a final fully connected layer to generate classification logits for activity category prediction.

### 3.3. Spatiotemporal Dynamic Residual Feature Encoder

This paper adopts a compact TS-ResNet encoder inspired by TS-DyConv for wearable sensor time-series representation learning. The encoder follows a residual learning paradigm and replaces the conventional fixed convolution in the residual blocks with a spatiotemporal dynamic convolution module, denoted as TS-Dynamic Conv2d. Different from a standard ResNet designed for image inputs, the proposed encoder is tailored to single-channel sensor time-series inputs, where the temporal dimension and the sensor-feature dimension jointly form a two-dimensional feature map. Residual connections are adopted to improve gradient propagation and stabilize feature learning.

Given an input sensor window $X_{0}\in R^{B\times 1\times T\times S}$, where *B*, *T*, and *S* denote the batch size, sliding-window length, and sensor-feature dimension, respectively, the initial feature extraction layer first maps the input to a 64-channel feature representation:

(2) $$X_{1}=\mathrm{MaxPool}\left(\delta \left(\mathrm{BN}\left({\mathrm{Conv}}_{7\times 7}\left(X_{0}\right)\right)\right)\right),$$

where ${\mathrm{Conv}}_{7\times 7}\left(\cdot \right)$ denotes a 2D convolution, BN(·) denotes batch normalization, and δ(·) is the ReLU activation function. The max-pooling operation reduces the feature-map resolution and suppresses local noise.

The compact TS-ResNet encoder contains three residual stages with channel widths of 64, 128, and 200. The original fourth 512-channel residual stage is removed to reduce model complexity. Each residual stage contains one basic residual block. For the *l*-th residual block, the output is formulated as

(3) $$X_{l+1}=\delta \left(F_{l}\left(X_{l}\right)+D_{l}\left(X_{l}\right)\right),$$

where $F_{l}\left(\cdot \right)$ denotes the residual transformation implemented by two TS-Dynamic Conv2d layers with batch normalization and ReLU activation, and $D_{l}\left(\cdot \right)$ denotes the identity or downsampling shortcut used to match the feature dimension when necessary.

For an intermediate feature map $X\in R^{B\times C\times T^{\prime }\times S^{\prime }}$, the TS-Dynamic Conv2d module first extracts a global context descriptor:

(4) $$z=\delta \left(\mathrm{BN}\left(W_{1}\mathrm{GAP}\left(X\right)\right)\right),$$

where GAP(·) denotes global average pooling over the temporal and sensor-feature dimensions, $W_{1}$ is a 1×1 convolutional mapping, BN(·) denotes batch normalization, and δ(·) is the ReLU activation function.

Based on the global descriptor *z*, the module generates two attention terms: the temporal/channel attention weight $\alpha_{t}$ and the spatial kernel attention weight $\alpha_{s}$:

(5) $$\alpha_{t}=\sigma \left(\frac{W_{t}z}{\tau }\right),\;\alpha_{s}=\sigma \left(\frac{W_{s}z}{\tau }\right),$$

where $W_{t}$ and $W_{s}$ are 1×1 convolutional mappings, τ is the temperature parameter, and σ(·) denotes the sigmoid function. Here, $\alpha_{t}\in R^{B\times C\times 1\times 1}$ recalibrates the feature channels, while $\alpha_{s}\in R^{B\times 1\times 1\times 1\times k\times k}$ modulates the spatial positions of the convolution kernel.

The input feature is first recalibrated by

(6) $$\tilde{X}=X⊙\alpha_{t},$$

where ⊙ denotes element-wise multiplication with broadcasting. Then, a sample-specific attention-modulated dynamic convolution kernel is constructed as

(7) $${\hat{W}}_{b}=\alpha_{s,b}⊙W,$$

where $W\in R^{C_{\mathrm{out}}\times C_{\mathrm{in}}\times k\times k}$ is the learnable convolution kernel, and *b* indexes the sample in the mini-batch. In the revised compact implementation, k=3—namely, a single attention-modulated 3×3 dynamic kernel is used. The output of TS-Dynamic Conv2d is computed as

(8) $$Y_{b}={\hat{W}}_{b}*{\tilde{X}}_{b},$$

where ∗ denotes convolution. Through this formulation, the encoder can adaptively recalibrate informative feature responses and adjust the effective convolutional kernel according to different sensor time-series patterns.

After the last residual stage, global average pooling is applied to obtain the final feature vector:

(9) $$h=\mathrm{GAP}(X_{\mathrm{out}}).$$

The feature vector *h* is then fed into the classifier for activity prediction. In addition, the same feature representation is used by the SimCLR projection head during contrastive representation learning, ensuring that the classification and contrastive branches are based on a consistent compact encoder output.

### 3.4. Multi-Objective Joint Optimization Loss Function

The training process of AWARE-Net constitutes a multi-task joint optimization paradigm, encompassing three strategic dimensions: contrastive learning, weighted classification loss, and decision smoothing.

#### 3.4.1. SimCLR-Based Contrastive Auxiliary Task

To enhance the robustness of learned features against noise, after the projection head outputs normalized feature vectors, we construct a similarity matrix and formulate a normalized temperature-scaled cross-entropy loss. Its mathematical formulation is defined as follows:

(10) $$\begin{array}{c} L_{SimCLR}=-\log\frac{\exp\left(\mathrm{sim}(z_{i},z_{j})/\tau \right)}{\sum_{k=1}^{2N}{⊮}_{\left[k\neq i\right]}\exp\left(\mathrm{sim}(z_{i},z_{k})/\tau \right)}, \end{array}$$

where $\mathrm{sim}(z_{i},z_{j})$ denotes the cosine similarity between feature vectors $z_{i}$ and $z_{j}$, τ represents the temperature hyperparameter, and ${⊮}_{\left[k\neq i\right]}$ is an indicator function that evaluates to 1 when k≠i and 0 otherwise.

#### 3.4.2. Class-Balanced Cross-Entropy Loss

To address the long-tailed distribution problem in sensor data, this paper adopts a class-balanced cross-entropy loss integrated with label smoothing. The class-balanced weight is calculated based on the effective number of samples. Assuming that the number of real samples for a certain category is $n_{i}$, its effective number of samples is defined as $1-\beta^{n_{i}}$. The class-balanced weight $\omega_{i}$ is given by

(11) $$\omega_{i}=\frac{1-\beta }{1-\beta^{n_{i}}}$$

After normalization, the weights are applied to the cross-entropy loss of each sample to increase the weights of minority classes and reduce the weights of majority classes. Meanwhile, label smoothing is introduced to avoid overfitting in model prediction:

(12) $$L_{CB-CE}=-\frac{1}{N}\sum_{i=1}^{N}\omega_{y_{i}}\left[\left(1-\varepsilon \right)\log\left(p_{y_{i}}\right)+\frac{\varepsilon }{C}\sum_{c=1}^{C}\log\left(p_{c}\right)\right]$$

where ε is the label smoothing coefficient, *C* is the number of activity categories, $p_{y_{i}}$ is the predicted probability of the ground-truth class, and $p_{c}$ is the predicted probability of category *c*.

#### 3.4.3. Total Variation Smooth Regularization

To alleviate local temporal feature fluctuations caused by sensor noise and sample imbalance, we apply total variation smooth regularization to the encoded temporal feature representation before global average pooling rather than to the final classification logits. TV regularization is computed along the temporal dimension of the encoded feature map within each sliding window.

Let $H\in R^{B\times T^{\prime }\times D}$ denote the temporal feature representation before global average pooling, where *B* is the batch size, $T^{\prime }$ is the temporal length after the encoder, and *D* denotes the feature dimension. The TV loss is defined as

(13) $$L_{\mathrm{TV}}=\frac{1}{B(T^{\prime }-1)D}\sum_{b=1}^{B}\sum_{t=1}^{T^{\prime }-1}{∥H_{b,t+1,:}-H_{b,t,:}∥}_{1}.$$

Here, $H_{b,t,:}$ denotes the encoded feature vector at the *t*-th temporal position within the *b*-th sliding window. The TV regularization is computed within each sliding window rather than between different windows. Therefore, the mini-batch does not need to contain temporally consecutive sliding windows. By constraining adjacent temporal features to vary smoothly, $L_{\mathrm{TV}}$ reduces local temporal jitter while preserving the discriminative temporal structure required for activity recognition.

#### 3.4.4. Final Training Objective

During the overall joint training process, we integrate the aforementioned supervised classification loss, contrastive learning auxiliary loss, and smooth regularization constraint into a unified global loss function for backpropagation optimization. The final loss function is defined as follows:

(14) $$L_{total}=\alpha \cdot L_{SimCLR}+L_{CB-CE}+\beta \cdot L_{TV}$$

In this formula, $L_{CB-CE}$ denotes the primary classification loss that incorporates the class-balanced mechanism and label smoothing. The hyperparameters α and β are used to balance the contributions of different tasks, representing the weight of contrastive learning and the weight of smooth regularization, respectively.

## 4. Experiments

In this section, we conduct experiments on three human activity recognition benchmark datasets to systematically validate the effectiveness of the proposed lightweight joint optimization framework AWARE-Net. We first introduce the experimental setup, including the adopted datasets, baseline and state-of-the-art comparison methods, evaluation metrics, and model implementation details. Subsequently, we present the comparative experimental results on the three datasets, which demonstrate that the recognition performance of the proposed method outperforms mainstream classic models and advanced state-of-the-art methods in the field, with significant performance superiority. Finally, a series of ablation experiments are carried out to verify the effectiveness of the core loss function and key optimization modules, and we analyze the impact of critical hyperparameters, including the contrastive loss weight and total variation regularization weight, on the performance of the proposed method.

### 4.1. Experimental Setup

#### 4.1.1. Datasets

We select three publicly available benchmark SHAR datasets in this study, including OPPORTUNITY [31], PAMAP2 [32], and USC-HAD [33]. These datasets cover diverse scenarios, including multi-modal sensing, complex interactive activities, and activities of daily living and fall detection, and exhibit the typical characteristic of class imbalance, making them well-suited for validating the robustness of our proposed method.

**OPPORTUNITY:** The OPPORTUNITY dataset is collected from 19 subjects performing 17 daily interactive activities, including cleaning, cooking, and door opening and closing. A total of four Inertial Measurement Units (IMUs) are mounted on the chest and limbs of the subjects, with data sampled at a frequency of 30 Hz.

**PAMAP2:** The PAMAP2 dataset is collected from nine participants. These participants wear three Inertial Measurement Units (IMUs) and one physiological sensor, and perform 12 activities including walking, running, cycling, jumping, and so on. The sampling rate is 100 Hz. This dataset covers free-living indoor and outdoor scenarios, with a relatively balanced class distribution.

**USC-HAD:** The USC-HAD dataset is collected via sensor-based acquisition. Fourteen subjects perform 12 categories of daily activities, with significant differences in individual attributes, including gender, age, height, and weight among these subjects. It contains a total of 12,409 samples, with a data sampling rate of 100 Hz, and it exhibits a high degree of class diversity.

In the experiment, we perform missing value imputation and denoising cleaning on the OPPORTUNITY and PAMAP2 datasets. The basic information of each dataset and the sliding window size and overlap rate parameters are presented in Table 1.

#### 4.1.2. Metrics

We utilize accuracy and macro-F_1_ scores [34] to evaluate the performance of the proposed model. Since the HAR task considered in this paper is a multi-class classification problem, accuracy is computed as the proportion of correctly classified samples:

(15) $$\mathrm{Accuracy}=\frac{1}{N}\sum_{j=1}^{N}I\left({\hat{y}}_{j}=y_{j}\right),$$

where *N* denotes the number of test samples, $y_{j}$ is the ground-truth label of the *j*-th sample, ${\hat{y}}_{j}$ is the predicted label, and I(·) is the indicator function.

For each activity class *i*, precision, recall, and F_1_ score are computed as

(16) $${\mathrm{Precision}}_{i}=\frac{TP_{i}}{TP_{i}+FP_{i}},\;{\mathrm{Recall}}_{i}=\frac{TP_{i}}{TP_{i}+FN_{i}},$$

(17) $$F_{1,i}=\frac{2\cdot {\mathrm{Precision}}_{i}\cdot {\mathrm{Recall}}_{i}}{{\mathrm{Precision}}_{i}+{\mathrm{Recall}}_{i}}.$$

Here, $TP_{i}$, $FP_{i}$, and $FN_{i}$ are computed for the *i*-th class in a one-vs-rest manner. The reported F_1_ score is the macro-F_1_, which averages the F_1_ scores of all classes equally:

(18) $$\mathrm{Macro}\text{-}F_{1}=\frac{1}{C}\sum_{i=1}^{C}F_{1,i},$$

where *C* denotes the number of activity classes. Additionally, we apply the confusion matrix to visualize the classification performance of the model.

#### 4.1.3. Baselines

We select two categories of representative models as our baselines to comprehensively evaluate our proposed method, including classic deep learning models commonly used in the field of human activity recognition and state-of-the-art mainstream methods with outstanding performance in sensor-based human activity recognition in recent years. Specifically, the classic deep learning models include models such as CNN [4], ResNet [18], and MobileNetV2 [19], as well as sequential learning models such as GRU [35] and LSTM [6]. In addition, the mainstream advanced methods cover MobileNetV2 [19], TS-DyConv [20], Dual-INABA [36], Channel Selection CNN [21], Dual Attention DanHAR [37], Multi-ResAtt [38], DWOSC [39], Innovative Dual-Decoupling [22], etc. The classical baselines and the main reproducible competitors are evaluated under the same preprocessing pipeline and experimental environment, while some state-of-the-art results are quoted from the corresponding publications when seed-wise predictions or complete reproducible implementations are unavailable.

To further clarify the novelty boundary of AWARE-Net with respect to recent HAR methods, Table 2 provides a component-level comparison. The proposed method does not claim novelty from a single isolated module. Instead, its main contribution lies in integrating a lightweight spatiotemporal encoder with class-balanced supervised learning, SimCLR-based contrastive auxiliary learning, and TV-based smoothness regularization into a unified reliability-aware framework. Compared with dynamic-convolution or attention-based methods, AWARE-Net additionally incorporates imbalance-aware and robustness-oriented joint optimization. Compared with contrastive-learning methods, AWARE-Net further considers class-balanced supervision, temporal smoothness, and lightweight inference for wearable-sensor HAR.

#### 4.1.4. Implementation Details

All models in our work are implemented using the PyTorch 2.0 deep learning framework, and all experiments are conducted on a server equipped with an NVIDIA RTX A6000 GPU with 48 GB of video memory. All models are trained using Adam as the optimizer. A step decay strategy is adopted for the learning rate, which drops to 50% of its initial value every 60 epochs. The parameter settings for each dataset are shown in Table 3. To evaluate the statistical stability of the experimental results, AWARE-Net and the main comparison methods were independently trained and evaluated five times with different random seeds under the same experimental protocol. The reported results are presented as mean (±) standard deviation.

### 4.2. Comparative Experiments

In this section, we conduct comparative experiments on three benchmark datasets against baseline models and existing state-of-the-art methods to comprehensively evaluate and verify the performance of our proposed method. For the compared methods, the F_1_ score is reported whenever it is available in the original publications; otherwise, it is marked as “×” because it cannot be reliably calculated without confusion matrices, per-class precision/recall, or prediction results.

#### 4.2.1. OPPORTUNITY Dataset Results

Table 4 shows the performance of our proposed AWARE-Net method on the OPPORTUNITY dataset, which consistently outperforms all baseline models.The overall recognition accuracy of the model on this dataset reaches 90.69±0.18%, with an F_1_ score of 86.23±0.25. The accuracy is higher than that of GRU at about 5.51%, LSTM at about 6.95%, ResNet at about 6.20%, and MobileNetV2 at about 5.95%. Further, the results show that our proposed method outperforms other existing state-of-the-art methods in the field, which is higher than that of the Channel Selection CNN at about 8.33%, Dual Attention DanHAR at about 7.94%, and TS-DyConv method at about 1.84%.

**Figure 2:**
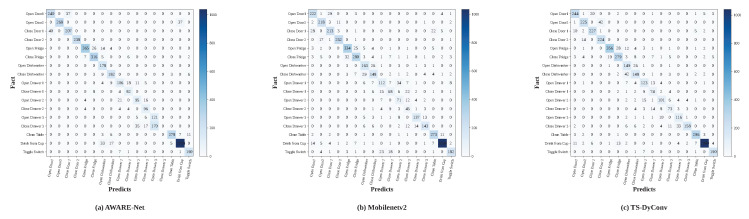
Confusion matrices on the OPPORTUNITY dataset.

To further analyze the performance of different models, Figure 2 depicts the confusion matrices we plotted to evaluate the prediction performance of MobileNetV2, TS-DyConv and AWARE-Net method on the OPPORTUNITY dataset. The number of correctly predicted samples for each activity is shown by the dark color blocks on the main diagonal. By comparing the three groups of confusion matrices, we can see that the main diagonal values of our proposed method for the vast majority of categories are significantly higher than those of the MobileNetV2 and TS-DyConv methods. Precisely, in the classification tasks of “open refrigerator” and “close refrigerator”, our proposed method correctly predicts 365 and 316 cases, respectively, while MobileNetV2 correctly predicts 334 and 280 cases, respectively, and TS-DyConv correctly predicts 356 and 279 cases, respectively. The classification accuracy of our proposed method outperforms these two types of models.

#### 4.2.2. PAMAP2 Dataset Results

**Figure 3:**
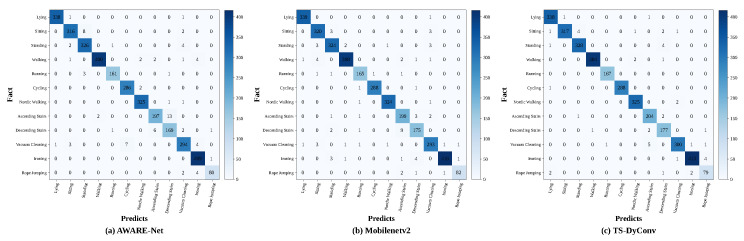
Confusion matrices on the PAMAP2 dataset.

Table 5 shows the performance of our proposed AWARE-Net method on the PAMAP2 dataset, which consistently outperforms all baseline models. The overall recognition accuracy of the model on this dataset reaches 98.38±0.12%, with an F_1_ score of 97.92±0.18%. The accuracy is higher than that of LSTM at about 3.90%, ResNet at about 0.57%, and MobileNetV2 at about 1.12%. Further, the results show that our proposed method outperforms other existing state-of-the-art methods in the field, which is higher than that of the Innovative Dual-Decoupling method at about 4.04%, TS-DyConv method at about 0.34%, and Dual-INABA at about 0.23%. In addition, Figure 3 shows the prediction results of the three types of methods on the PAMAP2 dataset via the confusion matrix. As shown in the figure, the diagonal values of our proposed method for most activities are consistently higher than those of the MobileNetV2 and TS-DyConv methods. Precisely, in the two tasks of “walking up stairs” and “walking down stairs”, our proposed method successfully predicts 204 and 177 samples, respectively, MobileNetV2 successfully predicts 197 and 169 samples, respectively, and TS-DyConv successfully predicts 199 and 175 samples, respectively.

#### 4.2.3. USC-HAD Dataset Results

**Figure 4:**
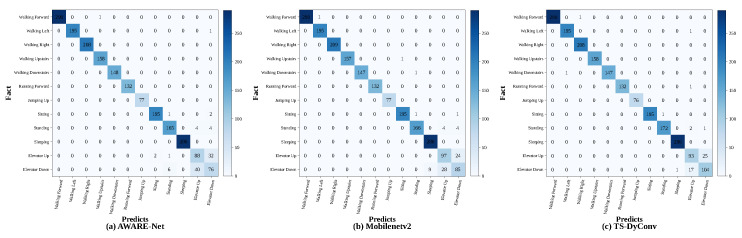
Confusion matrices on the USC-HAD dataset.

Table 6 shows the performance of all models on the USC-HAD dataset. The accuracy and F_1_ score of our proposed AWARE-Net reach 97.35±0.16% and 95.48±0.21%, respectively. Compared with the baseline models, the accuracy of AWARE-Net is improved by about 12.14% compared with LSTM, by about 3.49% compared with ResNet, and by about 1.25% compared with MobileNetV2. The performance of our proposed method is slightly lower than that of DWOSC. This is because the DWOSC method performs better in handling hard-to-classify samples, while the lightweight network used in this experiment has limitations in learning and representing long-range fine-grained features. In addition, the accuracy of the AWARE-Net method is improved by approximately 2.64% and 2.22% over Innovative Dual-Decoupling and TS-DyConv, respectively. In Figure 4, we visualize the prediction results of the three methods on this dataset using confusion matrices. Specifically, our proposed method outperforms MobileNetV2 and TS-DyConv in the classification tasks of “taking elevator upward” and “taking elevator downward”. For MobileNetV2, the number of misclassified samples between the two activities is as high as 40 and 32, respectively, while the TS-DyConv model also has 28 and 24 misclassified cases. In contrast, the number of misclassified samples of the AWARE-Net method is 17 and 25, respectively. However, when dealing with similar static behaviors, the proposed model shows recognition bias. For example, in the prediction of the “standing” activity, the proposed model misclassifies five samples of “standing” as “sleeping”.

#### 4.2.4. Robustness Evaluation Under Noisy Environments

**Figure 5:**
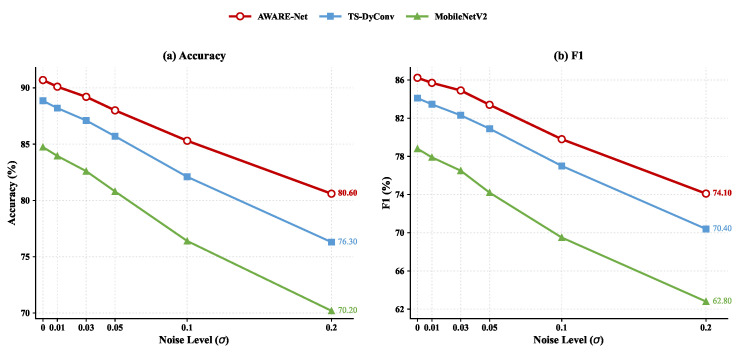
Robustness comparison under different Gaussian noise intensities on the OPPORTUNITY dataset.

To evaluate the robustness of the proposed method in noisy environments, we inject Gaussian noise with varying intensities into the normalized test set inputs of the OPPORTUNITY dataset, and compare the variation trends in accuracy and F1 scores of AWARE-Net, TS-DyConv, and MobileNetV2 under different noise levels. As can be seen from Figure 5, with the increase in noise intensity, the performance of all models degrades to varying degrees, while AWARE-Net exhibits the slowest overall degradation rate and maintains superior performance across the entire noise range. This result demonstrates that the dynamic feature encoding and joint optimization strategy introduced in this paper can effectively improve the model’s adaptability to noise disturbances, enabling it to maintain favorable discriminability and stability in complex sensor environments.

### 4.3. Analysis Experiments

In this part, we conduct the analysis study to show the efficiency of our proposed joint optimization framework, specifically evaluating the effectiveness of each module and loss function. Specifically, we compare the direct impact of different loss functions on the model’s classification performance. Then, based on the baseline model, we introduce class-balanced loss, contrastive auxiliary tasks, and temporal smoothing regularization to analyze the influence of each module on the final performance.

#### 4.3.1. Ablation Study on Loss Functions

In this subsection, we present a comprehensive evaluation of our proposed CB-CE loss function. Specifically, we compare it with the commonly used CE, FL, and CB loss on the OPPORTUNITY, PAMAP2, and USC-HAD datasets to verify and assess its generalization performance. The experimental results are shown in Table 7.

We can see from Table 7 that the CB-CE loss function outperforms other loss functions overall. CB-CE loss achieves an accuracy of 89.77% and an F_1_ score of 85.32% on the OPPORTUNITY dataset, which is significantly superior to other loss functions. On the PAMAP2 dataset, the CB-CE loss achieves an accuracy of 97.93% and an F_1_ score of 96.96%, also outperforming other loss functions. On the USC-HAD dataset, the CB-CE loss achieves an accuracy of 96.83% and an F_1_ score of 94.98%, which is also superior to other loss functions.

#### 4.3.2. Ablation Study of Key Modules

**Figure 6:**
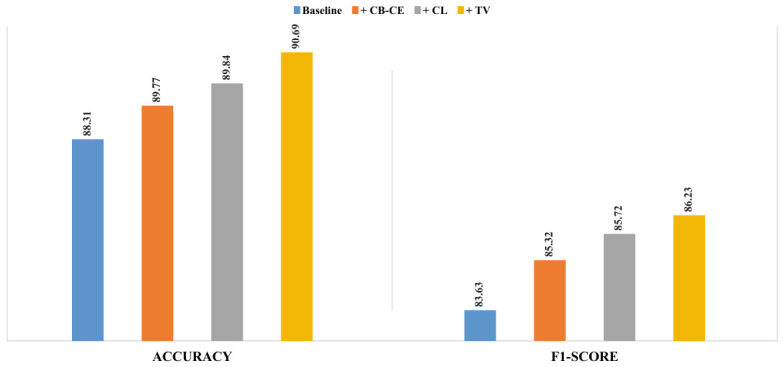
Ablation study of key modules on the OPPORTUNITY dataset.

In this section, we conduct ablation studies on the OPPORTUNITY dataset to verify the effectiveness of the optimization strategies proposed in this paper. It can be observed from the data in Figure 6 that, after adding the class-balanced loss to the baseline model, the model accuracy increased from 88.31% to 89.77%, with an F_1_ score of 85.32%. After further incorporating the contrastive auxiliary task into the model, the accuracy rose to 89.84% and the F_1_ score reached 85.72%. Finally, with the addition of the temporal smoothing regularization module, the model achieved optimal performance, with the accuracy climbing to 90.69% and the F_1_ score reaching 86.23%.Therefore, it is demonstrated that the class-balanced loss alleviates the problem of imbalanced sample distribution, the contrastive auxiliary task enhances feature robustness, and the temporal smoothing regularization effectively suppresses temporal jitter, thus improving the stability of continuous human activity recognition.

#### 4.3.3. Hyperparameter Analysis

In this section, we investigate the impact of two key hyperparameters in the AWARE-Net framework on model performance, namely the contrastive loss weight coefficient α and the TV regularization weight coefficient β. We set the values of α to 0.0, 0.05, 0.1, 0.2 and the values of β to 0.05, 0.1, 0.2, 0.3, resulting in a total of 16 hyperparameter configurations. The search ranges of α and β were selected according to the roles and relative strengths of the auxiliary loss terms. For α, the value of 0 removes the contrastive component, while 0.05, 0.1, and 0.2 correspond to weak, moderate, and relatively strong contrastive constraints. This range allows us to evaluate whether contrastive representation learning improves feature discrimination without overwhelming the supervised classification objective. For β, the values from 0.05 to 0.3 cover weak to relatively strong temporal regularization. Larger values were not included because excessive TV regularization may over-smooth the sensor sequence and weaken discriminative motion variations.

**Figure 7:**
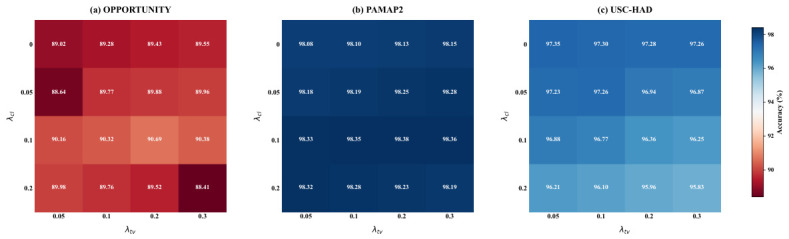
The hyperparameter study on the sequence learning pipeline.

Experiments were conducted on three datasets, OPPORTUNITY, PAMAP2, and USC-HAD, with the results presented in Figure 7.

It can be observed from the data in Figure 7 that, for the OPPORTUNITY and PAMAP2 datasets with complex action features and high sequence noise, the model achieved optimal performance on both datasets when the parameter combination was set to α = 0.1 and β = 0.2. Specifically, the model reached an accuracy of 90.69% and an F_1_ score of 86.23% on the OPPORTUNITY dataset and an accuracy of 98.38% and an F_1_-score of 97.92% on the PAMAP2 dataset. On the USC-HAD dataset, the model obtained optimal performance when α = 0 and β = 0.05, with an accuracy of 97.35% and an F_1_ score of 95.48%. The optimal values of (α) and (β) vary across datasets, indicating that the auxiliary loss terms are dataset-dependent. For OPPORTUNITY and PAMAP2, the best performance is achieved at (α = 0.1) and (β = 0.2), suggesting that moderate contrastive learning and temporal regularization are beneficial for complex and noisy sensor sequences. In contrast, USC-HAD obtains the best result at (α = 0) and (β = 0.05). This may be because USC-HAD contains relatively clearer activity patterns and lower sequence uncertainty, where the supervised classification loss is already sufficient to learn discriminative features. In such cases, the contrastive loss may impose unnecessary representation constraints and slightly reduce classification optimality. Therefore, the contrastive and TV terms should be regarded as adaptive auxiliary constraints: they are more useful for complex and noisy datasets, while weaker regularization is preferable for cleaner datasets with clearer class boundaries.

The above results indicate that moderately increasing the weight values of α and β can effectively improve the model’s accuracy on datasets with complex action features and high noise, while increasing these coefficients may lead to a downward trend in accuracy on other simple datasets with clear action boundaries. Therefore, it is demonstrated that a reasonable joint hyperparameter configuration can enhance the discriminability of latent space features and effectively suppress the high-frequency fluctuations in the temporal prediction process.

## 5. Discussion

**Figure 8:**
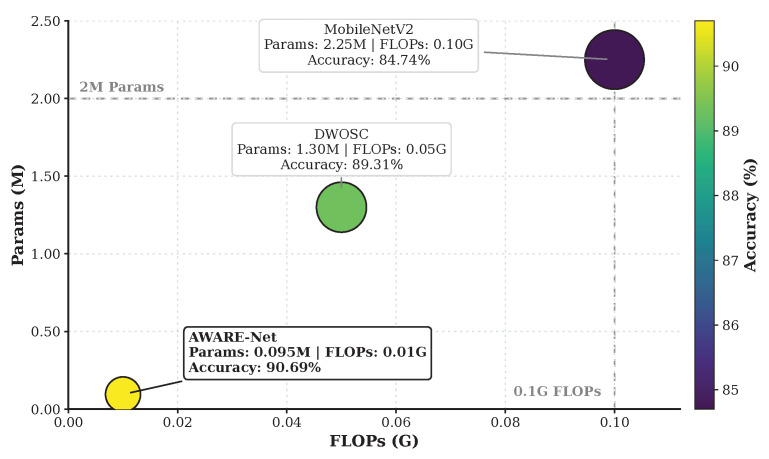
Lightweight performance comparison of different models.

In this section, we analyze the lightweight property and computational complexity of the proposed AWARE-Net framework. The comparison results of MobileNetV2, DWOSC, and AWARE-Net in terms of parameter count, computational complexity, and recognition performance are presented in Figure 8. To ensure a fair and clear complexity analysis, the model complexity of AWARE-Net is measured using the actual OPPORTUNITY input size of 1×1×30×113, where 30 is the sliding-window length and 113 is the sensor-feature dimension. The compact TS-ResNet encoder contains three residual stages with channel widths of 16, 32, and 64. The initial convolution is implemented as a lightweight 3×3 convolution, and the TS-Dynamic Conv2d module uses a single attention-modulated 3×3 dynamic convolution kernel. The per-module complexity breakdown of the deployed encoder-classifier model is shown in Table 8. The SimCLR-based contrastive branch and the two augmented views are used only during training. During inference, no augmented views are generated and the contrastive loss is not computed. The final prediction only relies on the encoder and classification head. Therefore, the contrastive auxiliary task introduces additional training costs but does not increase the inference cost of the deployed HAR model.

As shown in Figure 8, MobileNetV2 contains 2.25 M parameters and requires 0.10 G FLOPs, while DWOSC contains 1.30 M parameters and requires 0.05 G FLOPs. In contrast, the deployed AWARE-Net encoder–classifier model contains only approximately 0.095 M parameters and requires approximately 0.006 G FLOPs, which is rounded to 0.01 G FLOPs in the comparison figure. These results indicate that AWARE-Net has a substantially lower computational burden than the compared lightweight models. The above analysis demonstrates complexity-level potential suitability for resource-constrained edge deployment. Actual deployment performance, including hardware latency, memory footprint, and energy consumption, still depends on the target device, inference framework, and optimization strategy and will be further evaluated on representative wearable or embedded platforms in future work.

It is demonstrated that the proposed AWARE-Net joint framework improves the accuracy of human activity recognition, achieves significant optimization in parameter count and computational complexity, and possesses reliable deployment feasibility in practical scenarios, including health monitoring, sports activity tracking and fall detection.

## 6. Conclusions

In this paper, we address the core challenges of wearable sensor-based human activity recognition tasks, and propose a lightweight joint optimization framework named AWARE-Net. The proposed framework adopts TS-ResNet as its backbone and combines spatiotemporal dynamic convolution with a multi-objective joint loss function to simultaneously tackle the pain points of noise interference, data imbalance, and the trade-off between recognition accuracy and lightweight deployment. Experiments on the OPPORTUNITY, PAMAP2, and USC-HAD datasets show that AWARE-Net achieves accuracies of 90.69%, 98.38%, and 97.35%, respectively, achieves superior representative comparison methods while only using approximately 0.095M parameters and 0.006G FLOPs. Nevertheless, the current study is mainly validated on public offline datasets with fixed sensor configurations, and its performance under long-term real-world use, cross-device deployment, and missing-sensor conditions still requires further investigation. Future work will focus on cross-subject and cross-device generalization, adaptive sensing under incomplete or unreliable sensor inputs, lightweight Transformer-based temporal modeling, and hardware-aware optimization for real-time deployment on wearable edge platforms.

## Figures and Tables

**Table 1:** Summary statistics for the datasets.

Dataset	Activity	Frequencies	Window Size	Overlap	Train	Test
OPPORTUNITY	17	30 Hz	30	50%	70%	30%
PAMAP2	12	100 Hz	171	0	70%	30%
USC-HAD	12	100 Hz	512	50%	80%	20%

**Table 2:** Component-level comparison between AWARE-Net and representative modern HAR methods.

Method	Main Focus	Dynamic/ST Modeling	Attention/Fusion	Contrastive Learning	Class-Imbalance Loss	Smoothness Regularization	Unified Lightweight Optimization
TS-DyConv [20]	Temporal–spatial dynamic convolution for wearable HAR	Yes	Partial	No	No	No	Partial
CLEAR [13]	Multimodal contrastive feature refinement	No	Yes	Yes	No	No	Partial
DanHAR [37]	Dual attention modeling for multimodal HAR	No	Yes	No	No	No	No
Dual-INABA [36]	Inception–attention and recurrent feature modeling	Partial	Yes	No	No	No	No
DWOSC [39]	Dynamic weight optimization and smoothness constraint	Partial	No	No	Partial	Yes	Partial
**AWARE-Net**	Reliability-aware lightweight joint optimization	Yes	Yes	Yes	Yes	Yes	Yes

**Table 3:** Implementation details and optimal experimental settings.

Dataset	Training Setting	AWARE-Net-Specific Setting
OPPORTUNITY	lr = $2\times 10^{-3}$; wd = $1\times 10^{-6}$; bs = 128; epoch = 200; optimizer = Adam; scheduler = step decay, lr × 0.5 every 60 epochs.	Backbone = ResNet-8; channels = 64/128/256; projection hidden dim = 256; dropout = 0; $n_{\mathrm{views}}=2$; τ=0.07; $\beta_{\mathrm{CB}}=0.999$; focal γ=2.0; loss weights: $\lambda_{\mathrm{CB}}=1.0$, $\lambda_{\mathrm{CL}}=0.1$, $\lambda_{\mathrm{TV}}=0.2$.
PAMAP2	lr = $2\times 10^{-3}$; wd = $1\times 10^{-6}$; bs = 256; epoch = 400; optimizer = Adam; scheduler = step decay, lr × 0.5 every 60 epochs.	Backbone = ResNet-8; channels = 64/128/256; projection hidden dim = 256; dropout = 0; $n_{\mathrm{views}}=2$; τ=0.07; $\beta_{\mathrm{CB}}=0.999$; focal γ=2.0; loss weights: $\lambda_{\mathrm{CB}}=1.0$, $\lambda_{\mathrm{CL}}=0.1$, $\lambda_{\mathrm{TV}}=0.2$.
USC-HAD	lr = $1\times 10^{-3}$; wd = $1\times 10^{-6}$; bs = 256; epoch = 300; optimizer = Adam; scheduler = step decay, lr × 0.5 every 60 epochs.	Backbone = ResNet-8; channels = 64/128/256; projection hidden dim = 256; dropout = 0; $n_{\mathrm{views}}=2$; τ=0.07; $\beta_{\mathrm{CB}}=0.999$; focal γ=2.0; loss weights: $\lambda_{\mathrm{CB}}=1.0$, $\lambda_{\mathrm{CL}}=0$, $\lambda_{\mathrm{TV}}=0.05$.

**Table 4:** Accuracy (%) and F_1_ score of all models on the OPPORTUNITY dataset. Results are reported as mean ± standard deviation over five independent runs. The best results are bolded.

Model	Accuracy (%)	F_1_ Score
GRU	85.18±0.31	79.66±0.42
LSTM	83.74±0.28	76.55±0.39
ResNet	84.49±0.25	78.57±0.36
MobileNetV2	84.74±0.27	78.80±0.34
Channel Selection CNN	82.36	×
DanHAR	82.75	×
TS-DyConv	88.85±0.22	×
AWARE-Net	90.69±0.18	86.23±0.25

**Table 5:** Accuracy (%) and F_1_ score of all models on the PAMAP2 dataset. Results are reported as mean ± standard deviation over five independent runs when available. The best results are bolded.

Model	Accuracy (%)	F_1_ Score
GRU	95.78±0.24	94.05±0.31
LSTM	94.48±0.29	92.70±0.36
ResNet	97.31±0.18	96.73±0.22
MobileNetV2	96.76±0.21	96.69±0.25
Innovative Dual-Decoupling	94.34	×
TS-DyConv	98.04±0.15	×
Dual-INABA	98.15	×
AWARE-Net	98.38±0.12	97.92±0.18

**Table 6:** Accuracy (%) and F_1_ score of all models on the USC-HAD dataset. Results are reported as mean ± standard deviation over five independent runs when available. The best results are bolded.

Model	Accuracy (%)	F_1_ Score
GRU	93.40±0.26	91.41±0.35
LSTM	85.21±0.34	85.92±0.41
ResNet	93.86±0.22	92.04±0.30
MobileNetV2	96.10±0.18	94.76±0.24
Innovative Dual-Decoupling	94.71	×
TS-DyConv	95.13±0.20	×
DWOSC	97.67	×
AWARE-Net	97.35±0.16	95.48±0.21

**Table 7:** The accuracy (%) and F_1_ score of different loss functions on OPPORTUNITY, PAMAP2, and USC-HAD datasets. The best results are bolded.

	Dataset	OPPORTUNITY		PAMAP2		USC-HAD	
Loss		Accuracy (%)	F_1_ Score (%)	Accuracy (%)	F_1_ Score (%)	Accuracy (%)	F_1_ Score (%)
CE		88.31	83.63	97.81	96.85	96.42	94.34
FL		87.94	83.28	96.87	96.34	95.58	94.02
CB		88.56	84.09	97.86	96.89	96.61	94.63
CB-CE		**89.77**	**85.32**	**97.93**	**96.96**	**96.83**	**94.98**

**Table 8:** Per-module parameter and computational complexity breakdown of the deployed AWARE-Net model. FLOPs are measured with the OPPORTUNITY input size of 1×1×30×113.

Module	Output Size	Params	FLOPs(G)
Initial Conv-BN-ReLU	1×16×15×57	176	0.00025
TS-ResNet Layer1, 16 channels	1×16×8×29	6184	0.00214
TS-ResNet Layer2, 32 channels	1×32×4×15	17,800	0.00172
TS-ResNet Layer3, 64 channels	1×64×2×8	69,352	0.00184
Global average pooling	1×64×1×1	0	–
Classifier FC	1×17	1105	0.000002
**Inference model total**	–	94,617	0.006

## Data Availability

The raw data supporting the conclusions of this article will be made available by the authors on request.
